# Supplementary material for: Acceptance and compliance with micronutrient powder (MNP) among children aged 6–23 months in northern Nigeria
Source: PLOS Glob Public Health. 2022 Oct 17;2(10):e0000961. doi: 10.1371/journal.pgph.0000961 (PMC10022258; doi:10.1371/journal.pgph.0000961)
Supplement: S1 Dataset — (ZIP) [file pgph.0000961.s008.zip › S8_Dataset/In-depth Interviews.zip!/In-depth Interviews/040715_A_SO_ID_2_CG.pdf]

IN DEPTH INTERVIEW WITH CARE GIVER IN MUDUNGO COMMUNITY OF SONG LOCAL GOVERNMENT ADAMAWA STATE BY ISHAKU DAVID SARASO ON THE 4TH JULY, 2015 DURATION 53M 56S.

INTRODUCTION: 060715_A_6A_ID_CG_2_DOC

I: Describe for me the people in your immediate family living in your household.

R: We are living with my husband and our children, they are Mohammed, Ismaila, Hamisu and Hafi'u.

I: Tell me on specific gender and age the child 6-23 months

R: Abdulmumini has 9 months and he is a male child.

CHILDREN 6-23 MOTNHS

I: We have heard of food challenges for young infant children in this setting related to the seasons.

R: During raining seasons we have many different types of food but during dry season the food are not much compare to raining seasons, secondly in time of planting some time we don't have food to give the child.

I: Tell me how you cope with this food challenges

R: You know as a poor man whenever he go out with his cow for ploughing and get money, we will buy food so that we will eat with the family members.

I: Please tell me how this challenges affect infant and young child feeding.

R: It affect infant and young child feeding because the food it not enough to eat and sometimes we have enough food to eat.

I: One thing we heard in other interviews that caregivers often give their children 6-23months tea. Could you define tea?

R: The tea is usually made up of sugar, milk (cow bell), Lipton and hot water.

EXPERIENCE WITH MNP DURING TRIAL

I: Now I want us to talk about the MNP that you received. Could you tell me about your experience with the product.

R: The product is very good because when you give your child, he will be looking good and also make him to be eating food.

I: Tell me more about positive aspect of the experience with MNP

R: Before I start given him MNP, he don't use to be taken pap but now that he is taken MNP use to be taken pap

CHALLENGES/BARRIERS USING MNP

I: Tell me about general challenges you had during the MNP trial

R: When I start given the child MNP, he use to vomit but after sometime (3 to 4 days) when he is use to it he don't vomit any more.

I: Can you tell me specific barriers that made it hard to use the MNP appropriately.

R: The first 4 days that made him to vomit is the problem.

I: Some caregivers mentioned that it was hard to store the MNP without it being taken by others or eaten by rodents. Did you have any similar experience?

R: No

I: You made mentioned that the first 4 days of given MNP to the child he vomit what did you do to the child.

R: Nothing, it just stops like that

I: Some mothers indicated that it might be hard to remember to use MNP. Did you have a similar experience and what did you do to remember.

R: There is nothing like that just that if you made up your mind that is all.

FEEDING CHALLENGES AND RESPONSE TO MNP

I: Tell me how you feed him the MNP to the child usually

R: I use to feed him with maize pap with MNP in a content which is just like syrup cover. I mixed it with my hand and feed the child.

I: Tell me when do you usually give MNP to the child

R: I use to give him around 9:00am and sometimes around 1:00pm

I: How extent that the child likes or dislike the taste of the MNP

R: HE likes it because sometimes when he sees me with MNP he use to welcome me with his hands and when I maxed it, he collect and take it with his hand.

I: Tell me how consumption of MNP impacted the child's current diet

R: He is eating food not like before and when a child eat food, he don't take much breast milk

SHARING MNP

I: In other communities we noticed that sharing of MNP occurred; could you talk about any sharing of MNP when you used it?

R: No, we don't share MNP with anybody

I: Tell me any sharing MNP due to food shortage during lean season

R: No, there is no way that you will give it to somebody without asking the person that gives you the product

I: Tell me whether caregivers or fathers ate the MNP as a snack

R: No, I cannot do that

I: Will 60 sachets be enough for 60days?

R: Yes because everyday i will be given the child 1 sachet.

PERCEPTIONS of the MNP

I: Now that you have used MNP, could you talk about how you perceive it

R: It is very good I want to even ask for more

I: Tell me whether you see it more as a food, a medicine or something else

R: Medicine

I: Why

R: Because it change my child body, makes him to eat food

WILLINGNESS TO PAY FOR MNP

I: If MNP were cheaply on sale in this community will you purchase this product

R: Yes

I: Why

R: Because it has improves my child health

I: How much would you be willing to pay for each sachet?

R: I can pay N20 for each sachet because I have seen it importance.

SOCIAL MARKETING THE MNP

I: You are giving me great information, now please tell me some ways that you believe would be effective for promoting MNP to caregivers in the community so that they would comply with using it appropriately.

R: Caregivers should make sure they give their children MNP and make use of it appropriately, let them not collect the product and keep it without using it or they use for sometimes and stop using it.

I: Tell me some effective ways to promote MNP its appropriate use

R: Tell caregivers that it will make their children to be eating food and socking breast milk and also improve their child health so they should use it.

I: Please tell me who influences your decision regarding feeding your baby with the MNP?

R: It is the community health workers because I have trust in them

I: Tell me who influences your decisions regarding feeding your baby the MNP

R: It is my husband because when the community health workers gave us MNP I show him he say ok and when he came back from farm he use to ask me have I give the MNP to the child?

I: Where would be good place to disseminate health information related to MNP?

R: As you people came, we should be gather under a tree

I: Who in your community do you trust the most for receiving messages about infant and young child feeding.

R: The community health workers

I: What forms of media do you prefer for receiving MNP MESSAGES?

R: Radio

I: What ways do you preferred of receiving education regarding IYCF and MNP

R: Let all the caregivers gather together so that the community health worker will help educate them

I: What would be best community channels to distribute the product

R: Let it be through the community health workers

I: Why

R: Because this is not the first time of doing this type of work and it is related to their field of work.

I: I want to hear some specific messages you think would be effectively promote the product

R: MY advice to the caregivers is that they should collect this product and make use of it because it is very good for our children’s health

I: What messages should be used to explain MNP to caregivers.

R: The product built the body, make the child look good and be eating food.

FINAL THOUGHTS

I: Finally is there anything else that you can share about your experience with this product

R: First of all I want to thank the government for bringing a very good thing to us, I pray that the good Lord will bless them and increase their knowledge.
